# 2-Cyclo­hexyl-4-methyl­tetra­hydro­pyran-4-ol^1^

**DOI:** 10.1107/S1600536810015333

**Published:** 2010-04-30

**Authors:** Edward R. T. Tiekink, Alexandra Macedo, Edison P. Wendler, Alcindo A. Dos Santos, Julio Zukerman-Schpector

**Affiliations:** a Department of Chemistry, University of Malaya, 50603 Kuala Lumpur, Malaysia; b Department of Chemistry, Universidade Federal de São Carlos, 13565-905 São Carlos, SP, Brazil; c Instituto de Química, Universidade de São Paulo, São Paulo-SP, Brazil

## Abstract

In the title compound, C_12_H_22_O_2_, the 4-methyl­tetra­hydro­pyran-4-ol ring adopts a conformation close to that of a chair and with the two O atoms *syn*; the cyclo­hexyl group occupies an equatorial position and adopts a chair conformation. In the crystal packing, supra­molecular chains along the *b* axis are sustained by O—H⋯O hydrogen bonds. These are connected into undulating layers in the *ab* plane by C—H⋯O inter­actions.

## Related literature

For background to the solvent-free catalysed synthesis of tetra­hydro­pyran odorants, see: Macedo *et al.* (2010 ▶). For conformational analysis, see: Cremer & Pople (1975 ▶)
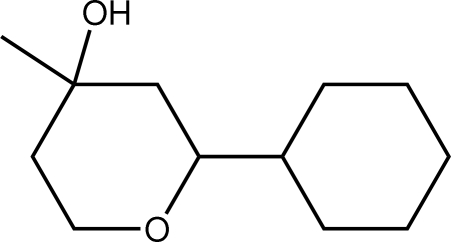


## Experimental

### 

#### Crystal data

C_12_H_22_O_2_
*M* *_r_* = 198.30
Orthorhombic, 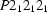

*a* = 5.5714 (10) Å
*b* = 11.0182 (12) Å
*c* = 18.753 (3) Å
*V* = 1151.2 (3) Å^3^
*Z* = 4
Mo *K*α radiation
μ = 0.08 mm^−1^
*T* = 153 K
0.20 × 0.10 × 0.08 mm

#### Data collection

Rigaku AFC12/SATURN724 diffractometer
Absorption correction: multi-scan (*ABSCOR*; Higashi, 1995 ▶) *T*_min_ = 0.510, *T*_max_ = 1.000
8308 measured reflections
1404 independent reflections
1338 reflections with *I* > 2σ(*I*)
*R*_int_ = 0.040

#### Refinement

*R*[*F* ^2^ > 2σ(*F* ^2^)] = 0.047
*wR*(*F* ^2^) = 0.107
*S* = 1.18
1404 reflections
128 parameters
H-atom parameters constrained
Δρ_max_ = 0.17 e Å^−3^
Δρ_min_ = −0.18 e Å^−3^

### 

Data collection: *CrystalClear* (Rigaku/MSC, 2005 ▶); cell refinement: *CrystalClear*; data reduction: *CrystalClear*; program(s) used to solve structure: *SIR97* (Altomare *et al.*, 1999 ▶); program(s) used to refine structure: *SHELXL97* (Sheldrick, 2008 ▶); molecular graphics: *ORTEP-3*( Farrugia, 1997 ▶) and *DIAMOND* (Brandenburg, 2006 ▶); software used to prepare material for publication: *publCIF* (Westrip, 2010 ▶).

## Supplementary Material

Crystal structure: contains datablocks global, I. DOI: 10.1107/S1600536810015333/su2176sup1.cif

Structure factors: contains datablocks I. DOI: 10.1107/S1600536810015333/su2176Isup2.hkl

Additional supplementary materials:  crystallographic information; 3D view; checkCIF report

## Figures and Tables

**Table 1:** Hydrogen-bond geometry (Å, °)

*D*—H⋯*A*	*D*—H	H⋯*A*	*D*⋯*A*	*D*—H⋯*A*
O2—H1*o*⋯O1^i^	0.92	1.88	2.773 (2)	164
C13—H13b⋯O2^ii^	0.98	2.40	3.337 (3)	159
C6—H6b⋯O2^ii^	0.99	2.58	3.552 (3)	168

Symmetry codes: (i) 
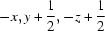
; (ii) 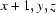.

**Fig. 1.**
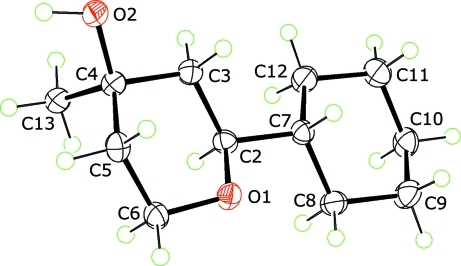
The molecular structure of (I) showing the atom-labelling scheme and displacement ellipsoids at the 50% probability level.

**Fig. 2.**
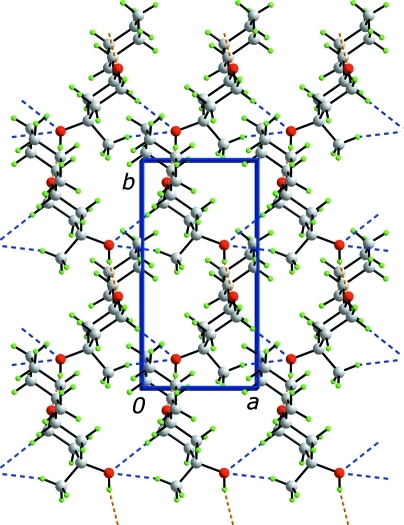
A view of a supramolecular layer in (I) in the ab plane. The O–H···O hydrogen bonds and C–H···O contacts are shown as orange and blue dashed lines, respectively. Colour code: O, red; C, grey; and H, green.

**Fig. 3.**
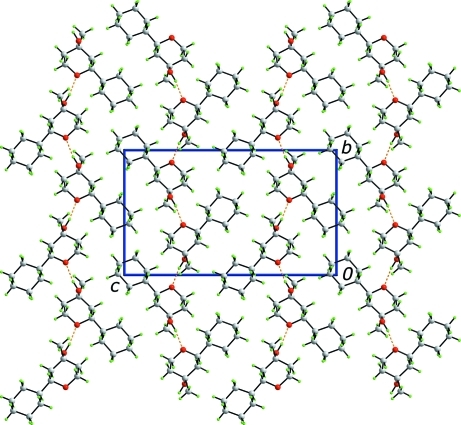
Stacking of undulating layers in (I) along the c direction. The O–H···O hydrogen bonds are shown as orange dashed lines. Colour code: O, red; C, grey; and H, green.

## supplementary crystallographic information

### Comment

The structure of the title compound, (I), was investigated as a part of a study into the solvent-free catalysed synthesis of tetrahydropyran odorants (Macedo *et al.*, 2010). The key observation in the molecular structure is the *syn* relationship of the oxygen atoms. The six-membered 4-methyltetrahydropyran-4-ol ring adopts a conformation close to a chair as defined by the ring-puckering parameters of q_2_ = 0.041 (2) Å, q_3_ = -0.559 (2) Å, Q = 0.560 (2) Å, θ = 176.6 (2) °, and φ_2_ = 159 (3) ° (Cremer & Pople, 1975). The cyclohexyl substituent occupies an equatorial position and adopts an almost perfect chair conformation. In the crystal packing, O–H···O hydrogen bonding leads to the formation of supramolecular chains along the *b* axis, Table 1. These are connected by C–H···O contacts into a 2-D array in the *ab* plane, Fig. 2 and Table 1. The layers have an undulating topology and the pendent cyclohexyl rings inter-digitate along the *c* axis, Fig. 3.

### Experimental

The preparation and characterisation is as described in the literature (Macedo *et al.*, 2010). The compound was dissolved in a mixture of hexane:ethyl acetate (2:1) and left to stand for five days at room temperature. The white irregular crystals were collected and washed with a small amount of hexane before drying in air.

### Refinement

The H atoms were geometrically placed (O—H = 0.92 Å and C—H = 0.98-1.00 Å) and refined as riding with *U*_iso_(H) = 1.2*U*_eq_(C, O) or 1.5*U*_eq_(methyl-C). In the absence of significant anomalous scattering effects, 1934 Friedel pairs were averaged in the final refinement.

### Crystal data

**Table:**

C_12_H_22_O_2_	*F*(000) = 440
*M**_r_* = 198.30	*D*_x_ = 1.144 Mg m^−^^3^
Orthorhombic, *P*2_1_2_1_2_1_	Mo *K*α radiation, λ = 0.71073 Å
Hall symbol: P 2ac 2ab	Cell parameters from 3569 reflections
*a* = 5.5714 (10) Å	θ = 2.9–30.1°
*b* = 11.0182 (12) Å	µ = 0.08 mm^−^^1^
*c* = 18.753 (3) Å	*T* = 153 K
*V* = 1151.2 (3) Å^3^	Prism, colourless
*Z* = 4	0.20 × 0.10 × 0.08 mm

### Data collection

**Table:**

Rigaku AFC12K/SATURN724 diffractometer	1404 independent reflections
Radiation source: fine-focus sealed tube	1338 reflections with *I* > 2σ(*I*)
graphite	*R*_int_ = 0.040
ω scans	θ_max_ = 26.5°, θ_min_ = 2.9°
Absorption correction: multi-scan (*ABSCOR*; Higashi, 1995)	*h* = −5→6
*T*_min_ = 0.510, *T*_max_ = 1.000	*k* = −13→13
8308 measured reflections	*l* = −23→23

### Refinement

**Table:**

Refinement on *F*^2^	Primary atom site location: structure-invariant direct methods
Least-squares matrix: full	Secondary atom site location: difference Fourier map
*R*[*F*^2^ > 2σ(*F*^2^)] = 0.047	Hydrogen site location: inferred from neighbouring sites
*wR*(*F*^2^) = 0.107	H-atom parameters constrained
*S* = 1.18	*w* = 1/[σ^2^(*F*_o_^2^) + (0.0449*P*)^2^ + 0.288*P*] where *P* = (*F*_o_^2^ + 2*F*_c_^2^)/3
1404 reflections	(Δ/σ)_max_ < 0.001
128 parameters	Δρ_max_ = 0.17 e Å^−^^3^
0 restraints	Δρ_min_ = −0.18 e Å^−^^3^

### Special details

**Table:**

Geometry. All s.u.'s (except the s.u. in the dihedral angle between two l.s. planes) are estimated using the full covariance matrix. The cell s.u.'s are taken into account individually in the estimation of s.u.'s in distances, angles and torsion angles; correlations between s.u.'s in cell parameters are only used when they are defined by crystal symmetry. An approximate (isotropic) treatment of cell s.u.'s is used for estimating s.u.'s involving l.s. planes.
Refinement. Refinement of *F*^2^ against ALL reflections. The weighted *R*-factor wR and goodness of fit *S* are based on *F*^2^, conventional *R*-factors *R* are based on *F*, with *F* set to zero for negative *F*^2^. The threshold expression of *F*^2^ > 2σ(*F*^2^) is used only for calculating *R*-factors(gt) etc. and is not relevant to the choice of reflections for refinement. *R*-factors based on *F*^2^ are statistically about twice as large as those based on *F*, and *R*- factors based on ALL data will be even larger. In the absence of significant anomalous scattering effects, 967 Friedel pairs were averaged in the final refinement.

### Fractional atomic coordinates and isotropic or equivalent isotropic displacement parameters (Å^2^)

**Table:**

	*x*	*y*	*z*	*U*_iso_*/*U*_eq_	
O1	0.2884 (3)	0.09963 (12)	0.27691 (8)	0.0230 (4)	
O2	−0.2092 (3)	0.37416 (14)	0.28439 (9)	0.0274 (4)	
H1o	−0.2118	0.4452	0.2586	0.033*	
C2	0.2556 (4)	0.15401 (18)	0.34614 (11)	0.0208 (5)	
H2A	0.3980	0.2060	0.3573	0.025*	
C3	0.0318 (4)	0.23271 (19)	0.34544 (12)	0.0230 (5)	
H3A	0.0170	0.2740	0.3921	0.028*	
H3B	−0.1105	0.1799	0.3394	0.028*	
C4	0.0323 (4)	0.32838 (19)	0.28640 (12)	0.0220 (5)	
C5	0.0907 (4)	0.26595 (19)	0.21548 (12)	0.0233 (5)	
H5A	−0.0463	0.2143	0.2011	0.028*	
H5B	0.1142	0.3283	0.1781	0.028*	
C6	0.3154 (5)	0.18839 (19)	0.22109 (12)	0.0241 (5)	
H6A	0.3446	0.1468	0.1751	0.029*	
H6B	0.4555	0.2408	0.2314	0.029*	
C7	0.2457 (5)	0.04850 (19)	0.39950 (11)	0.0212 (5)	
H7	0.1038	−0.0030	0.3872	0.025*	
C8	0.4706 (5)	−0.0307 (2)	0.39425 (12)	0.0262 (6)	
H8A	0.4827	−0.0643	0.3454	0.031*	
H8B	0.6142	0.0202	0.4028	0.031*	
C9	0.4676 (5)	−0.1351 (2)	0.44794 (12)	0.0313 (6)	
H9A	0.3330	−0.1905	0.4367	0.038*	
H9B	0.6190	−0.1817	0.4441	0.038*	
C10	0.4400 (5)	−0.0879 (2)	0.52343 (12)	0.0296 (6)	
H10A	0.5826	−0.0390	0.5363	0.036*	
H10B	0.4299	−0.1572	0.5569	0.036*	
C11	0.2151 (5)	−0.0102 (2)	0.53033 (12)	0.0307 (6)	
H11A	0.2066	0.0238	0.5792	0.037*	
H11B	0.0716	−0.0616	0.5229	0.037*	
C12	0.2135 (5)	0.0940 (2)	0.47612 (11)	0.0281 (6)	
H12A	0.3446	0.1514	0.4877	0.034*	
H12B	0.0596	0.1385	0.4798	0.034*	
C13	0.2059 (5)	0.43185 (19)	0.30228 (12)	0.0246 (5)	
H13A	0.2006	0.4911	0.2633	0.037*	
H13B	0.3691	0.3995	0.3067	0.037*	
H13C	0.1595	0.4715	0.3470	0.037*	

### Atomic displacement parameters (Å^2^)

**Table:**

	*U*^11^	*U*^22^	*U*^33^	*U*^12^	*U*^13^	*U*^23^
O1	0.0312 (9)	0.0196 (7)	0.0183 (7)	0.0014 (7)	0.0004 (7)	−0.0011 (6)
O2	0.0215 (8)	0.0250 (8)	0.0358 (9)	0.0037 (7)	0.0021 (8)	0.0072 (7)
C2	0.0233 (12)	0.0215 (10)	0.0174 (10)	−0.0009 (10)	0.0015 (9)	−0.0014 (8)
C3	0.0250 (12)	0.0218 (10)	0.0222 (10)	0.0013 (10)	0.0021 (10)	0.0005 (9)
C4	0.0217 (12)	0.0204 (10)	0.0238 (10)	0.0006 (9)	0.0013 (10)	−0.0003 (9)
C5	0.0275 (12)	0.0213 (10)	0.0211 (11)	−0.0026 (10)	−0.0024 (10)	0.0014 (9)
C6	0.0290 (13)	0.0225 (10)	0.0208 (10)	−0.0008 (10)	0.0033 (11)	0.0019 (9)
C7	0.0230 (13)	0.0209 (10)	0.0198 (10)	0.0001 (10)	0.0004 (10)	0.0012 (8)
C8	0.0273 (14)	0.0278 (12)	0.0237 (11)	0.0054 (11)	0.0027 (11)	0.0017 (9)
C9	0.0364 (15)	0.0278 (12)	0.0297 (12)	0.0092 (12)	0.0004 (12)	0.0044 (10)
C10	0.0347 (15)	0.0285 (12)	0.0257 (11)	0.0028 (12)	−0.0031 (11)	0.0076 (10)
C11	0.0387 (15)	0.0330 (12)	0.0205 (10)	0.0016 (13)	0.0024 (12)	0.0030 (10)
C12	0.0371 (15)	0.0262 (11)	0.0209 (10)	0.0052 (12)	0.0037 (11)	0.0016 (9)
C13	0.0247 (12)	0.0210 (10)	0.0281 (11)	0.0006 (10)	0.0032 (10)	−0.0010 (9)

### Geometric parameters (Å, °)

**Table:**

O1—C2	1.441 (2)	C7—H7	1.0000
O1—C6	1.440 (2)	C8—C9	1.529 (3)
O2—C4	1.438 (3)	C8—H8A	0.9900
O2—H1O	0.9200	C8—H8B	0.9900
C2—C3	1.519 (3)	C9—C10	1.516 (3)
C2—C7	1.535 (3)	C9—H9A	0.9900
C2—H2A	1.0000	C9—H9B	0.9900
C3—C4	1.529 (3)	C10—C11	1.524 (4)
C3—H3A	0.9900	C10—H10A	0.9900
C3—H3B	0.9900	C10—H10B	0.9900
C4—C13	1.524 (3)	C11—C12	1.533 (3)
C4—C5	1.532 (3)	C11—H11A	0.9900
C5—C6	1.519 (3)	C11—H11B	0.9900
C5—H5A	0.9900	C12—H12A	0.9900
C5—H5B	0.9900	C12—H12B	0.9900
C6—H6A	0.9900	C13—H13A	0.9800
C6—H6B	0.9900	C13—H13B	0.9800
C7—C8	1.530 (3)	C13—H13C	0.9800
C7—C12	1.532 (3)		
			
C2—O1—C6	112.68 (15)	C2—C7—H7	108.0
C4—O2—H1O	109.1	C9—C8—C7	112.20 (19)
O1—C2—C3	109.48 (17)	C9—C8—H8A	109.2
O1—C2—C7	106.08 (15)	C7—C8—H8A	109.2
C3—C2—C7	114.10 (18)	C9—C8—H8B	109.2
O1—C2—H2A	109.0	C7—C8—H8B	109.2
C3—C2—H2A	109.0	H8A—C8—H8B	107.9
C7—C2—H2A	109.0	C10—C9—C8	110.99 (19)
C2—C3—C4	113.48 (18)	C10—C9—H9A	109.4
C2—C3—H3A	108.9	C8—C9—H9A	109.4
C4—C3—H3A	108.9	C10—C9—H9B	109.4
C2—C3—H3B	108.9	C8—C9—H9B	109.4
C4—C3—H3B	108.9	H9A—C9—H9B	108.0
H3A—C3—H3B	107.7	C9—C10—C11	110.8 (2)
O2—C4—C13	109.66 (17)	C9—C10—H10A	109.5
O2—C4—C3	105.02 (18)	C11—C10—H10A	109.5
C13—C4—C3	112.05 (18)	C9—C10—H10B	109.5
O2—C4—C5	109.49 (18)	C11—C10—H10B	109.5
C13—C4—C5	111.76 (19)	H10A—C10—H10B	108.1
C3—C4—C5	108.63 (17)	C10—C11—C12	111.7 (2)
C6—C5—C4	111.56 (18)	C10—C11—H11A	109.3
C6—C5—H5A	109.3	C12—C11—H11A	109.3
C4—C5—H5A	109.3	C10—C11—H11B	109.3
C6—C5—H5B	109.3	C12—C11—H11B	109.3
C4—C5—H5B	109.3	H11A—C11—H11B	107.9
H5A—C5—H5B	108.0	C11—C12—C7	112.08 (18)
O1—C6—C5	110.25 (18)	C11—C12—H12A	109.2
O1—C6—H6A	109.6	C7—C12—H12A	109.2
C5—C6—H6A	109.6	C11—C12—H12B	109.2
O1—C6—H6B	109.6	C7—C12—H12B	109.2
C5—C6—H6B	109.6	H12A—C12—H12B	107.9
H6A—C6—H6B	108.1	C4—C13—H13A	109.5
C8—C7—C12	110.03 (19)	C4—C13—H13B	109.5
C8—C7—C2	111.11 (18)	H13A—C13—H13B	109.5
C12—C7—C2	111.57 (17)	C4—C13—H13C	109.5
C8—C7—H7	108.0	H13A—C13—H13C	109.5
C12—C7—H7	108.0	H13B—C13—H13C	109.5
			
C6—O1—C2—C3	59.9 (2)	O1—C2—C7—C8	56.2 (2)
C6—O1—C2—C7	−176.59 (18)	C3—C2—C7—C8	176.83 (18)
O1—C2—C3—C4	−54.8 (2)	O1—C2—C7—C12	179.4 (2)
C7—C2—C3—C4	−173.46 (18)	C3—C2—C7—C12	−60.0 (3)
C2—C3—C4—O2	167.77 (17)	C12—C7—C8—C9	54.8 (3)
C2—C3—C4—C13	−73.3 (2)	C2—C7—C8—C9	178.84 (19)
C2—C3—C4—C5	50.7 (2)	C7—C8—C9—C10	−56.7 (3)
O2—C4—C5—C6	−165.23 (17)	C8—C9—C10—C11	56.2 (3)
C13—C4—C5—C6	73.1 (2)	C9—C10—C11—C12	−55.4 (3)
C3—C4—C5—C6	−51.1 (2)	C10—C11—C12—C7	54.6 (3)
C2—O1—C6—C5	−61.8 (2)	C8—C7—C12—C11	−53.5 (3)
C4—C5—C6—O1	57.0 (2)	C2—C7—C12—C11	−177.3 (2)

### Hydrogen-bond geometry (Å, °)

**Table:**

*D*—H···*A*	*D*—H	H···*A*	*D*···*A*	*D*—H···*A*
O2—H1o···O1^i^	0.92	1.88	2.773 (2)	164
C13—H13b···O2^ii^	0.98	2.40	3.337 (3)	159
C6—H6b···O2^ii^	0.99	2.58	3.552 (3)	168

Symmetry codes: (i) −*x*, *y*+1/2, −*z*+1/2; (ii) *x*+1, *y*, *z*.
